# Supplementary material for: Inflammation-like environments limit the loss of quorum sensing in Pseudomonas aeruginosa
Source: mSystems. 2025 Jul 7;10(8):e01722-24. doi: 10.1128/msystems.01722-24 (PMC12363187; doi:10.1128/msystems.01722-24)
Supplement: Supplemental material — Supplemental figures and tables. [file msystems.01722-24-s0001.pdf]

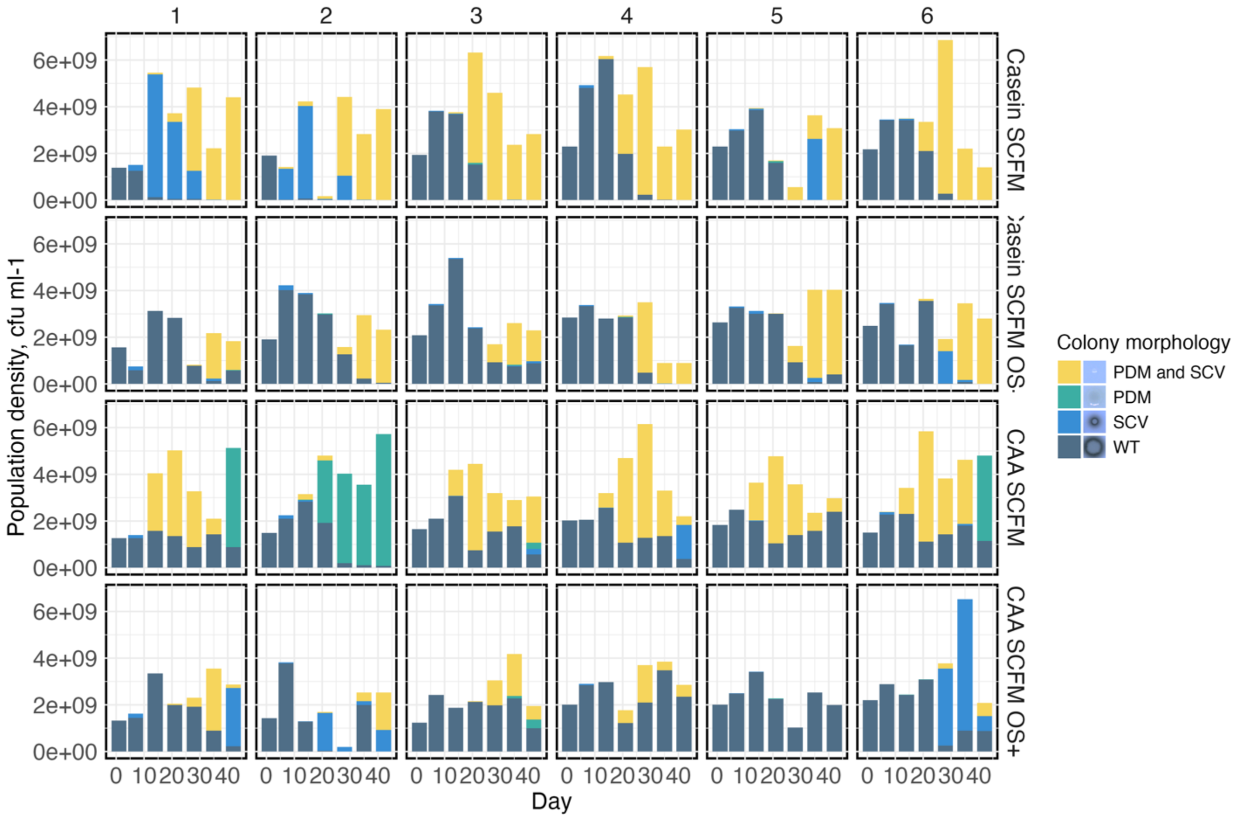

Figure S1. Colony morphology in different selection environments mimicking the lung environments. Four classes of colony morphologies were observed and examples are shown, similar to wild-type PA14 (WT), protease-deficient mutant (PDM), small colony variant (SCV), and small colony variant with a protease-deficiency (PDM and SCV).

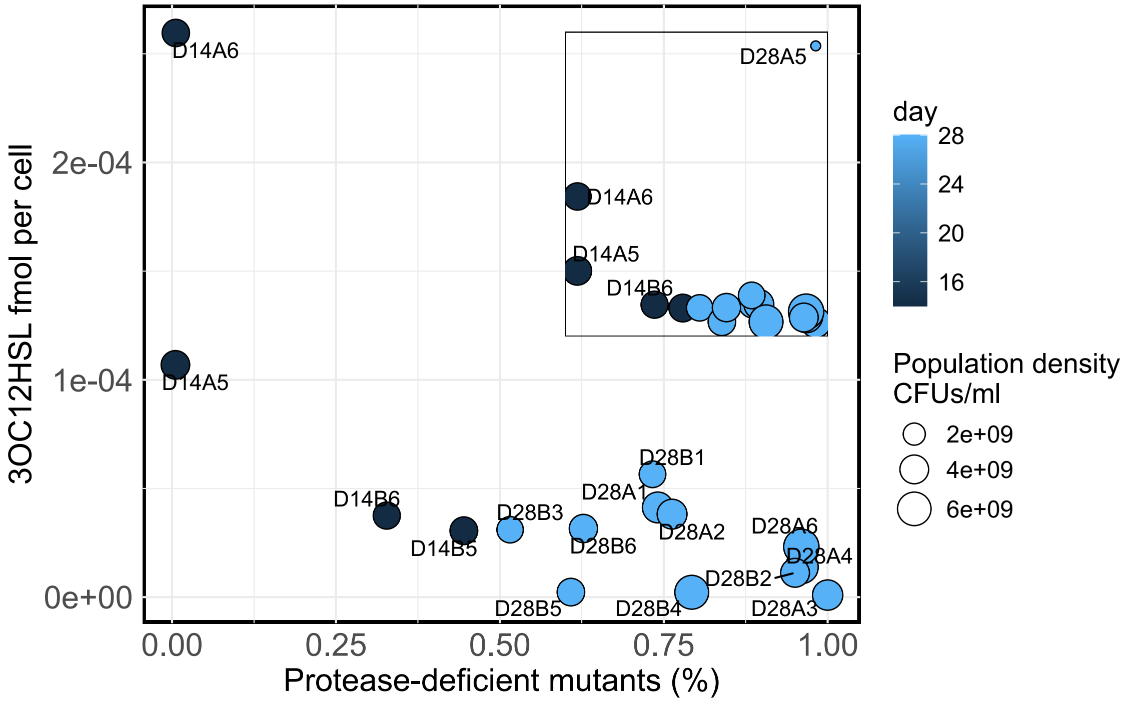

Figure S2. The average level of $3OC_{12}HSL$ per cell, measured by reporter plasmid (pTetR-LasR-pLuxR-GFP) divided by cell density measured by plate assay, in different samples negatively correlate with PDMs proportion (robust mixed effect model, log 10 ($3OC_{12}HSL$ fmol) per cell, PDMs proportion: $\beta = -1.03$, $SE = 0.43$, $t = -2.413$, 95% CI [-1.87, -0.19]). Dots are labeled as sampling timepoint following letter "D", then A for Casein SCFM, B for CAA SCFM and followed by population replicate number. Dot size correlates with population density and colored as sample collection date. To measure the association prior to and during the emergence of PDMs, 4 populations on day 14 and 12 populations on day 28 from the selective environments Casein SCFM and CAA SCFM were revived, sampled and measured.

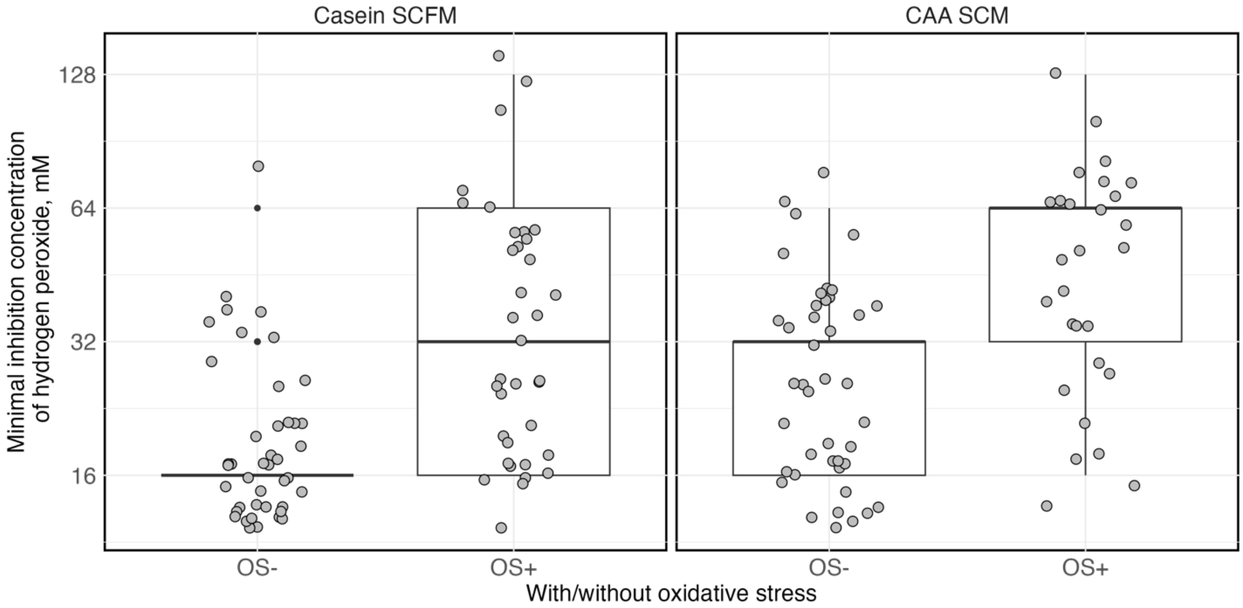

Figure S3. Minimal inhibition concentration of hydrogen peroxide of evolved PA14 from replicated populations under different selective environments (*n*=8 per population, 6 populations per selective environments, GLMM, count varied by interaction term between OS and MIC level, in CAA SCFM: $\chi^2_1 = 20.96$, $p < 0.001$; in Casein SCFM: $\chi^2_1 = 19.86$, $p < 0.001$).

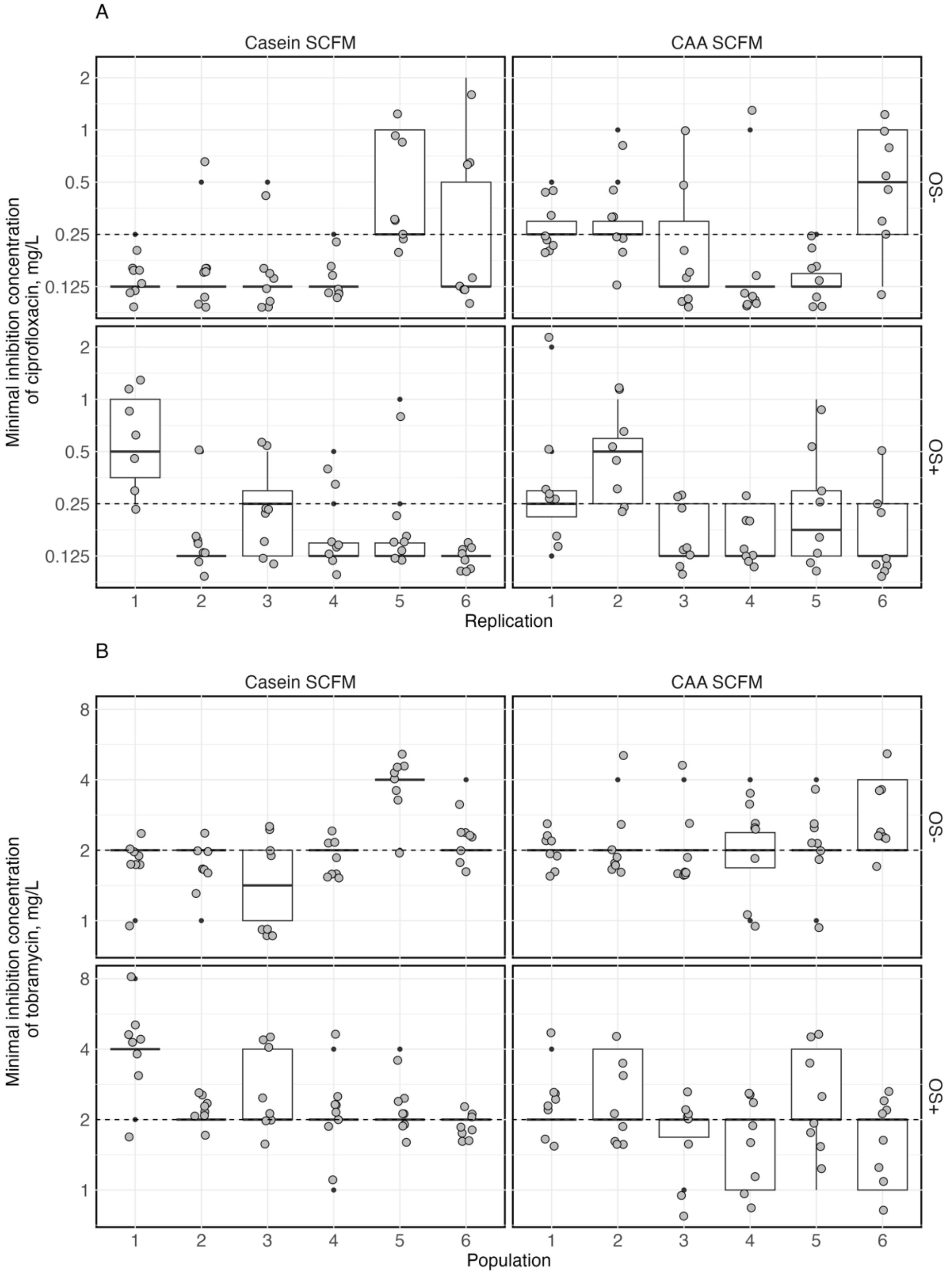

Figure S4. Minimal inhibition concentration (MIC) of ciprofloxacin (A) and tobramycin (B) of PA14 evolved in replicated populations under different selective environments. The MICs of ancestral PA14 are shown as dashed lines. Grey dots represent the average MIC of individual clones (*n* = 8

clones per populations, across 2 technical replicates), with a slight positional jitter applied to prevent overlap.

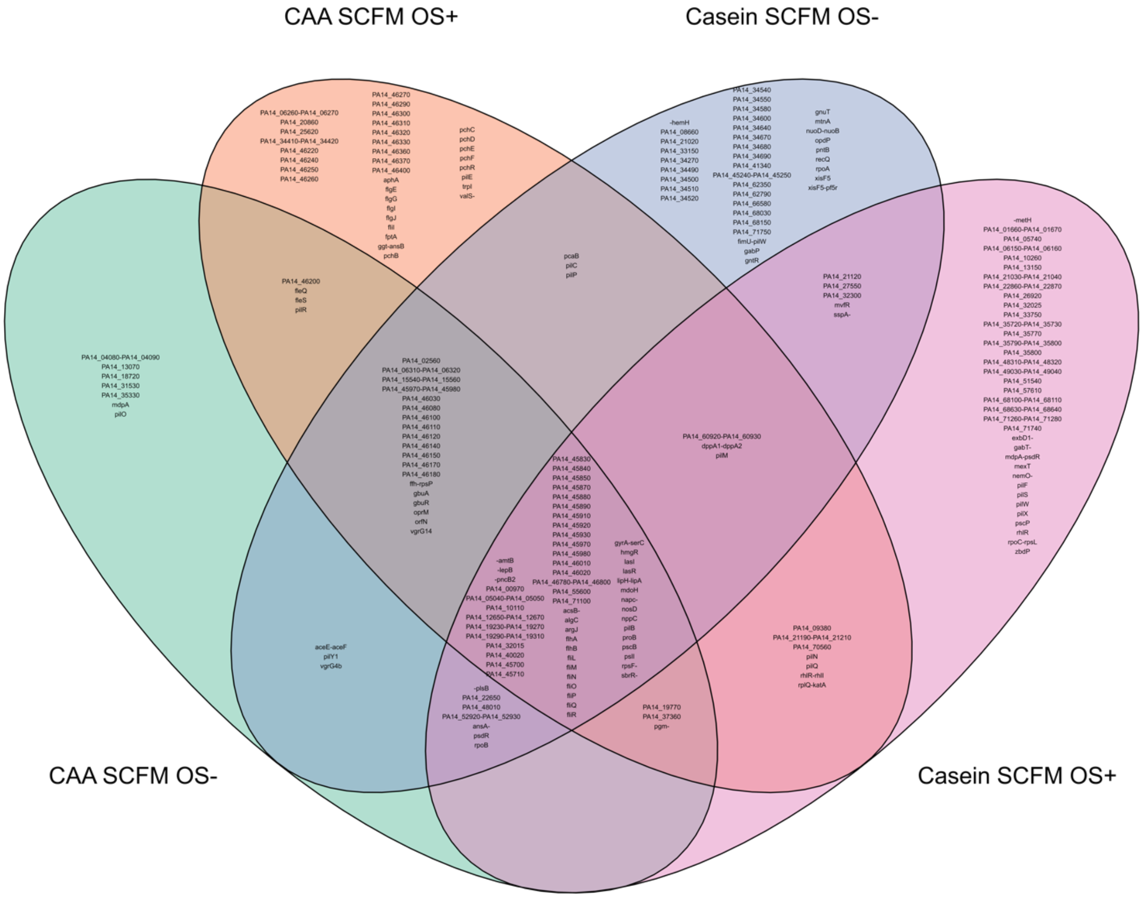

Figure S5. Non-synonymous mutations, indels and deletions in shared and unique genes or intergenic regions across four selective environments. All genes with functional mutations and intergenic regions with mutations observed on day 14, 28 and 42 are shown.

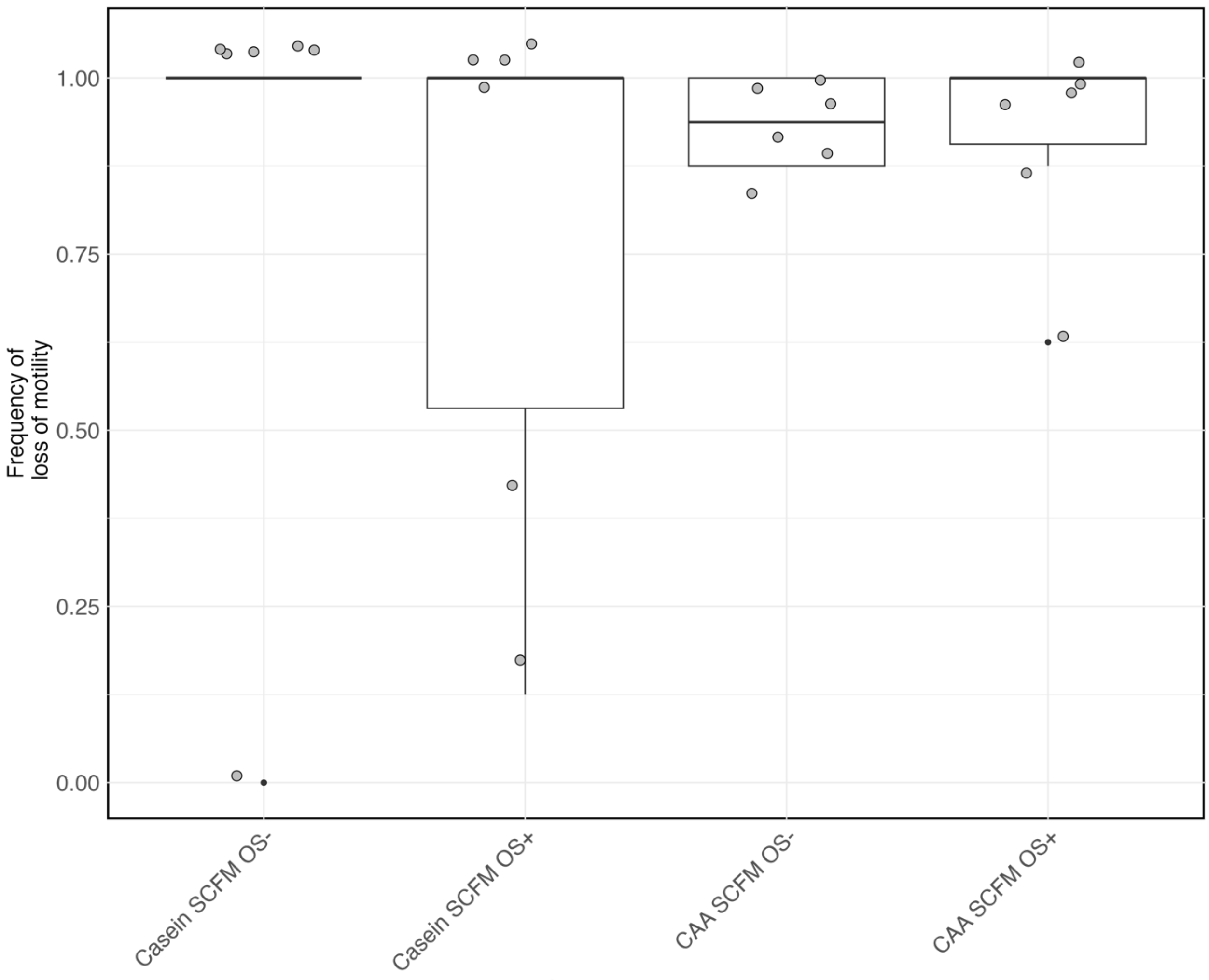

Figure S6. Loss of flagellar swimming motility across different selective environments on day 42. Box plot shows the distribution and average of, and grey points shows individual values of frequency of loss of flagellar motility in a replicate population (*n* = 8, per population).

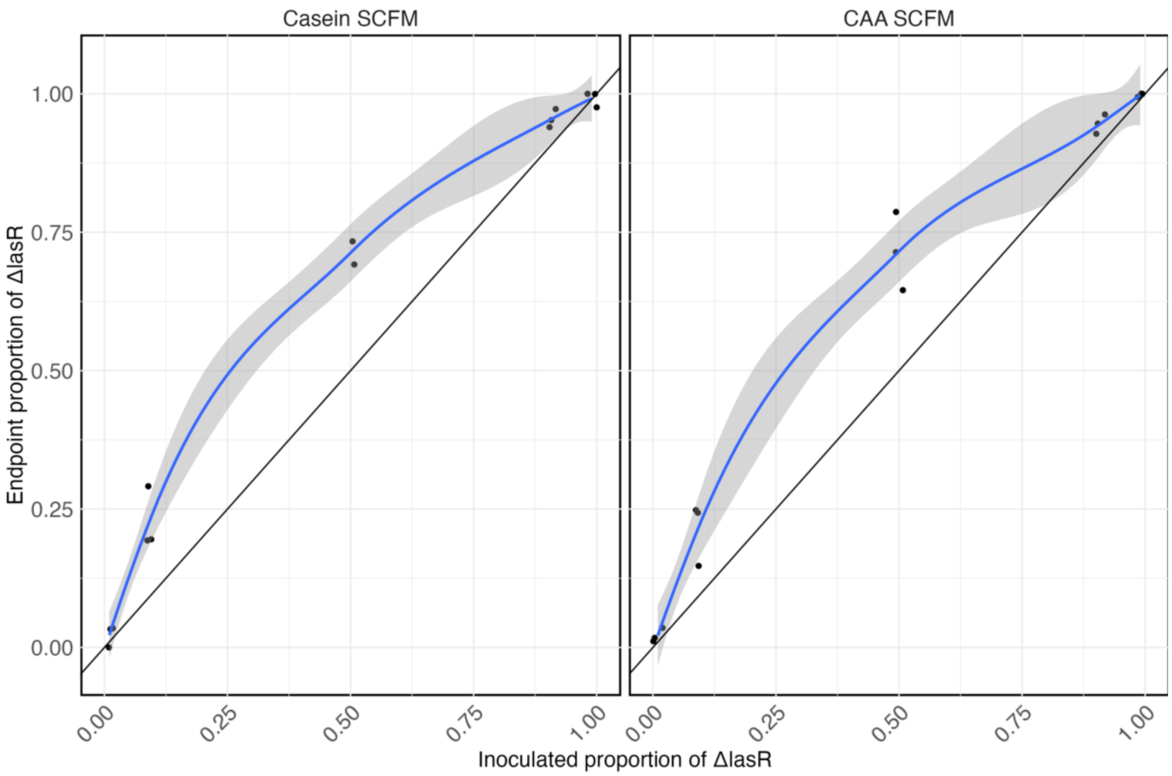

Figure S7. Competition assay between PA14 and PA14*ΔlasR* across starting ratios and nutrient conditions. The endpoint after 24 hours of co-culture and the inoculated proportions of PA14*ΔlasR* are shown (3 biological replicates per inoculated proportion of PA14*ΔlasR*).

Table S1. Synonymous SNPs and small indels across all evolved lines (6 replicated populations per selective environments, 4 selective environments) and 3 timepoints (days 14, 28, and 42).

Locus Label	Total Hits	Unique Hits	Locus Label	Total Hits	Unique Hits
PA14_02560	3	1	*PA14_61200*	44	2
PA14_21020	1	1	*PA14_65860*	66	1
PA14_24780	17	1	*argJ*	2	1
PA14_31720	5	1	*glgA*	3	1
PA14_34270	3	3	*mmsB*	1	1
PA14_46100	1	1	*nirB*	6	1
PA14_47880	25	1	*nppC*	30	1
PA14_48890	1	1	*rpoB*	4	4
PA14_55600	8	3			

Table S2. Nonsynonymous SNPs and small indels across all evolved lines (6 replicated populations per selective environments, 4 selective environments) and 3 timepoints (days 14, 28, and 42).

Locus Label	Total Hits	Unique Hits	Locus Label	Total Hits	Unique Hits
PA14_00970	17	1	*flgI*	2	1
PA14_02560	5	1	*flgJ*	2	2
PA14_05740	1	1	*flhA*	2	1
PA14_08660	4	4	*flhB*	2	1
PA14_09380	5	3	*fliI*	2	1
PA14_10110	37	1	*fliP*	1	1
PA14_10260	1	1	*fptA*	2	1
PA14_13070	2	1	*gabP*	1	1
PA14_13150	1	1	*hmgR*	15	1
PA14_18720	2	1	*lasR*	15	8
PA14_19770	3	1	*mdoH*	15	1
PA14_20860	1	1	*mdpA*	2	1
PA14_21020	1	1	*mexT*	1	1
PA14_21120	2	1	*mtnA*	1	1
PA14_22650	11	1	*mvfR*	3	3
PA14_25620	1	1	*nosD*	26	1
PA14_26920	1	1	*nppC*	7	2
PA14_27550	2	1	*opdP*	3	3
PA14_31530	1	1	*oprM*	3	1
PA14_32015	36	3	*orfN*	5	1
PA14_32025	1	1	*pcaB*	4	1
PA14_32300	3	1	*pchR*	2	1
PA14_33150	2	1	*pilB*	11	8
PA14_33750	1	1	*pilC*	3	3
PA14_34270	1	1	*pilE*	1	1
PA14_35330	2	1	*pilF*	2	1
PA14_35770	3	2	*pilM*	7	6
PA14_35800	2	2	*pilN*	3	2

Continued on next page

Table S2 – continued from previous page

Locus Label	Total Hits	Unique Hits	Locus Label	Total Hits	Unique Hits
PA14_37360	3	1	*pilO*	1	1
PA14_40020	37	1	*pilP*	2	2
PA14_41340	2	2	*pilQ*	4	3
PA14_46020	1	1	*pilR*	2	2
PA14_46100	1	1	*pilS*	1	1
PA14_48010	4	1	*pilW*	1	1
PA14_51540	4	4	*pilX*	1	1
PA14_55600	46	3	*pilY1*	2	2
PA14_57610	2	1	*pntB*	1	1
PA14_62350	1	1	*proB*	47	1
PA14_62790	5	5	*pscB*	58	1
PA14_66580	2	1	*pscP*	3	1
PA14_68030	5	5	*psdR*	28	18
PA14_68150	3	3	*pslI*	11	1
PA14_70560	22	11	*recQ*	5	2
PA14_71100	11	1	*rhlR*	3	2
PA14_71740	1	1	*rpoA*	2	1
PA14_71750	2	2	*rpoB*	8	4
algC	60	1	*trpI*	1	1
argJ	21	1	*vgrG14*	11	2
fleQ	31	6	*vgrG4b*	4	1
fleS	4	2	*xisF5*	8	4
flgE	2	1	*zbdP*	1	1
flgG	2	1			

Table S3. Hits in intergenic region across all evolved lines (6 replicated populations per selective environments, 4 selective environments) and 3 timepoints (days 14, 28, and 42).

Locus Label	Total Hits	Unique Hits	Locus Label	Total Hits	Unique Hits
-amtB	26	1	*PA14_60920-PA14_60930*	3	2
-hemH	1	1	*PA14_68100-PA14_68110*	1	1
-lepB	40	1	*PA14_68630-PA14_68640*	1	1
-metH	6	3	*PA14_71260-PA14_71280*	1	1
-plsB	11	3	*aceE-aceF*	2	1
-pncB2	8	1	*acsB-*	10	1
PA14_01660-PA14_01670	2	2	*ansA-*	9	2
PA14_04080-PA14_04090	1	1	*dppA1-dppA2*	3	1
PA14_05040-PA14_05050	14	1	*exbD1-*	1	1
PA14_06150-PA14_06160	3	3	*ffh-rpsP*	4	1
PA14_06260-PA14_06270	2	1	*fimU-pilW*	1	1
PA14_06310-PA14_06320	10	2	*gabT-*	2	1
PA14_12650-PA14_12670	24	1	*ggt-ansB*	1	1

Continued on next page

Table S3 – continued from previous page

**Locus Label**	**Total Hits**	**Unique Hits**	**Locus Label**	**Total Hits**	**Unique Hits**
PA14_15540-PA14_15560	3	1	*gyrA-serC*	50	1
PA14_19230-PA14_19270	16	1	*lipH-lipA*	20	1
PA14_19290-PA14_19310	68	1	*mdpA-psdR*	1	1
PA14_21030-PA14_21040	1	1	*napc-*	38	1
PA14_21190-PA14_21210	3	1	*nemO-*	2	2
PA14_22860-PA14_22870	1	1	*nuoD-nuoB*	1	1
PA14_34410-PA14_34420	2	1	*pgm-*	3	1
PA14_35720-PA14_35730	2	2	*rhlR-rhlI*	2	2
PA14_35790-PA14_35800	1	1	*rplQ-katA*	32	3
PA14_45240-PA14_45250	2	1	*rpoC-rpsL*	2	2

Continued on next page

Table S3 – continued from previous page

**Locus Label**	**Total Hits**	**Unique Hits**	**Locus Label**	**Total Hits**	**Unique Hits**
PA14_45970-PA14_45980	4	2	*rpsF-*	60	4
PA14_46780-PA14_46800	21	1	*sbrR-*	12	2
PA14_48310-PA14_48320	1	1	*sspA-*	4	1
PA14_49030-PA14_49040	2	2	*valS-*	1	1
PA14_52920-PA14_52930	6	2	*xisF5-pf5r*	6	3

Table S4. Deletions across all evolved lines (6 replicated populations per selective environments, 4 selective environments) and 3 timepoints (days 14, 28, and 42).

Locus Label	Total Hits	Locus Label	Total Hits
pchB	2	*PA14_45890*	27
pchC	2	*PA14_45910*	27
pchD	2	*PA14_45920*	27
pchR	2	*PA14_45930*	27
pchE	2	*lasI*	27
pchF	2	*lasR*	27
PA14_34490	1	*PA14_45970*	27
PA14_34500	1	*PA14_45980*	25
PA14_34510	1	*PA14_46010*	19
PA14_34520	1	*PA14_46020*	17
PA14_34540	1	*PA14_46030*	15
PA14_34550	1	*gbuR*	14
PA14_34580	1	*gbuA*	14
PA14_34600	1	*PA14_46080*	14
gnuT	1	*PA14_46100*	13
PA14_34640	1	*PA14_46110*	9
gntR	1	*PA14_46120*	7
PA14_34670	1	*PA14_46140*	7
PA14_34680	1	*PA14_46150*	7
PA14_34690	1	*PA14_46170*	7
flhA	8	*PA14_46180*	7
PA14_45700	14	*PA14_46200*	5
PA14_45710	16	*PA14_46220*	2
flhB	17	*aphA*	2
fliR	20	*PA14_46240*	2
fliQ	20	*PA14_46250*	2
fliP	23	*PA14_46260*	2
fliO	25	*PA14_46270*	2
fliN	26	*PA14_46290*	2

Continued on next page

Table S4 – continued from previous page

Locus Label	Total Hits	Locus Label	Total Hits
fliM	27	*PA14_46300*	2
fliL	27	*PA14_46310*	2
PA14_45830	27	*PA14_46320*	2
PA14_45840	27	PA14_46330	2
PA14_45850	27	PA14_46360	2
PA14_45870	27	PA14_46370	1
PA14_45880	27	PA14_46400	1
PA14_45840	27	PA14_46330	2
PA14_45850	27	PA14_46360	2
PA14_45870	27	PA14_46370	1
PA14_45880	27	PA14_46400	1
